# Feasibility, efficacy, and cautionary note of endoscopic resection for gastric tube cancer after esophagectomy

**DOI:** 10.1007/s00464-022-09240-8

**Published:** 2022-05-23

**Authors:** Yasuhiro Inokuchi, Mamoru Watanabe, Kei Hayashi, Yoshihiro Kaneta, Mitsuhiro Furuta, Nozomu Machida, Shin Maeda

**Affiliations:** 1 Department of Gastroenterology, Kanagawa Cancer Center, 2-3-2 Asahi-ku, Nakao, Yokohama, Kanagawa 241-8515 Japan; 2 Department of Gastroenterology, Yokohama City University, Yokohama, Kanagawa 236-0004 Japan

**Keywords:** Endoscopic resection, Endoscopic submucosal dissection, Early gastric cancer, Gastric tube cancer, Esophagectomy, Metachronous

## Abstract

**Background:**

Gastric tube cancer (GTC), whose usual histology is adenocarcinoma, occurs frequently as a result of improved survival after esophagectomy. Whether endoscopic resection (ER) for GTC is safe and suitable and guidelines for treatment and follow-up remains unclear.

**Methods:**

Patients with GTC who underwent ER at Kanagawa Cancer Center Hospital between 1997 and 2020 were studied retrospectively to evaluate clinical characteristics and short- and long-term outcomes.

**Results:**

Twenty-two consecutive patients with 43 lesions were treated in 42 sessions of ER. Lesions were discovered at a median of 9.0 (0–21.8) years after esophageal surgery. Nine (40.9%) patients had multiple lesions at the time of the initial ER session. However, six (54.5%) of the 11 co-existing lesions were overlooked. The location of the middle third was an estimated risk factor for overlooking (*p* = 0.028). In endoscopic submucosal dissection (ESD) cases, the en bloc dissection rate was as high as 97.1%, and the rates of bleeding, perforation, and aspiration pneumonitis were 17.6%, 0%, and 2.9%, respectively. The bleeding rate was relatively higher than that in usual gastric ESD. Twelve patients (54.5%) experienced synchronous and/or metachronous multiple GTCs during their life span. Thirteen (61.9%) patients died during the median follow-up period of 5.9 (0.7–15.5) years. One patient (7.7%) died of GTC recurrence, 15.4 years after the initial non-curative ER date; 3 (23.1%) patients died of esophageal cancer recurrence, and 3 (23.1%) died of other organ malignancies. The 5-year overall survival rate was 85.0%, and the 5-year disease-specific survival rate was 100%.

**Conclusions:**

ER is feasible for GTCs. However, the rate of bleeding was high in ESD cases. Life-long endoscopic screening of metachronous lesions is desirable. Care should be taken not to overlook lesions in the middle third of the gastric tube. Early detection of esophageal cancer recurrence and other organ malignancies may improve prognosis.

Early gastric cancer (EGC) is restricted to the mucosa and submucosal layers [1]. EGCs currently account for more than 60% of all detected cases of gastric cancer, and they are being detected in increasing numbers in Japan [2–4]. Gastric tube cancer (GTC), which is gastric cancer whose usual histology is adenocarcinoma that develops in the stomach after esophagectomy, has also increased recently [5–8]. Surgical treatment of GTC requires removal of the entire reconstructed gastric tube; thus, this approach is very invasive, with high morbidity and mortality [9]. Therefore, for early-stage GTC, endoscopic resection (ER), including endoscopic mucosal resection (EMR) and endoscopic submucosal dissection (ESD), is frequently preferentially selected over surgical gastric tubectomy [10–12].

EMR is a conventional endoscopic method used to resect small, elevated lesions without ulceration [13]. On the other hand, ESD is an innovative technique developed to enable the resection of lesions without limitations in shape or size, regardless of the presence of ulceration and tumor location [10, 14–17]. Although both of these endoscopic procedures are minimally invasive and can usually be performed without any morbidity, ER, especially ESD for GTC, is technically difficult for endoscopists compared to ER for EGC in a normal stomach. The reasons for this are that the gastric tube is severely influenced by heart beats; it has a narrow lumen with an unusual fluid-pooling area and residual food, and when the lesion is located upon the suture line, severe fibrosis and staples are also present [18]. As a result, there are only a few reports concerning ER for GTC, and most of these previous reports involved only a small number of patients with a short follow-up duration [9, 18–27]. Additionally, there is no consensus on the duration of surveillance after endoscopic resection. Therefore, we retrospectively investigated the feasibility of ER for GTC and the long-term natural course after treatment in a large number of cases with a long follow-up period to help guide ER management in patients with GTC.

## Patients and methods

### Patients

We retrospectively investigated patients with GTC in the reconstructed gastric tube after esophagectomy for esophageal cancer who underwent ER at Kanagawa Cancer Center Hospital from 1997 to 2020. There were 22 patients with 43 lesions involving metachronous GTCs. Synchronous GTC was defined as lesions detected at the same time as the initial lesion or initially overlooked lesions that had not been detected with the initial lesion but were discovered within 1 year after ER for the initial lesion.

Surgically resected esophageal cancers of these patients were all squamous cell carcinoma, and the depth of invasion was intramucosal in five cases, submucosal in five, muscularis propria (MP) in three, deeper than MP in eight, and unknown in one. Eleven of them were revealed to have at least one lymph node metastasis in resected specimens, and none of them had distant metastasis at the point of ER for GTC.

This study was approved by the Research Ethics Committee of the Kanagawa Cancer Center, which complies with the International Guidelines for Ethical Review of Epidemiological Studies. Written informed consent for endoscopic treatment was provided by all recruited patients before each treatment. We also uploaded study information on the Kanagawa Cancer Center website to allow patients to withdraw from the study.

### Pretreatment

Around-the-lesion biopsy (i.e., biopsy performed to confirm the absence of tumor cells (using a microscope) outside the macroscopically determined margin) was performed beforehand to confirm lesion margins when the area of tumor invasion was unclear. On the day of ER, the margin was re-identified using white light endoscopy, chromoendoscopy with indigo carmine solution, and narrow-band imaging. Then, the perimeter of the lesion was marked using small multiple cautery units made by the tip of a high-frequency snare to clarify the range. The high-frequency generators used were ICC200 or VIO300D (ERBE Elektromedizin GmbH, Tübingen, Germany).

### EMR procedure

After a submucosal injection of normal saline was performed to lift the mucosal layer, conventional EMR or EMR using a ligation device was performed as previously reported [28]. Against a large lesion, EMR was repeated until the mucosa within the marked area had been totally resected.

### ESD procedure

Submucosal injection was performed to lift the mucosal layer, using glycerol (10% glycerol and 5% fructose, Chugai Pharmaceutical Co., Tokyo, Japan) or MucoUp (0.4% sodium hyaluronate; Johnson & Johnson, New Brunswick, New Jersey, USA) with a small amount of indigo carmine as the injection solution. A circumferential mucosal incision and submucosal dissection were performed using a needle knife (IT Knife 2) and DualKnife (Olympus Optical Co., Ltd., Tokyo, Japan).

### Short-term outcomes

The short-term outcomes included the en bloc resection rate, the rate of adverse events, and the curability rate evaluated according to histopathological assessment. Curability was determined according to the Japanese Gastric Cancer Association Gastric Cancer Treatment Guidelines 2010 (ver. 3) [29]. A curative resection was defined as satisfying all the following conditions: en bloc resection, negative horizontal and vertical margins, no lymphovascular infiltration, and an absolute or expanded indication for ER. Differentiated-type intramucosal cancers ≤ 20 mm in size without ulceration were categorized as lesions of absolute indication. The expanded indications were as follows: differentiated-type intramucosal cancers > 20 mm in size without ulceration, differentiated-type intramucosal cancers ≤ 30 mm in size with ulceration, differentiated-type submucosal superficial cancers ≤ 30 mm in size, and undifferentiated type intramucosal cancers ≤ 20 mm in size without ulceration (Table 1). Resection was judged as non-curative when at least one of these listed conditions was not satisfied. In addition, non-curative resection was divided into two groups: (1) non-curative resection with a possible risk of lymph node metastasis (LNM) and (2) non-curative with only a positive/inconclusive horizontal margin (HM1/HMX) or fractional resection.

**Table 1:** Expanded indications for ER

Depth	Ulceration	Histology	
		Differentiated	Undifferentiated
Intramucosal	Negative	> 20 mm	≤ 20 mm
	Positive	≤ 30 mm	–
Submucosal		≤ 30 mm	–

*ER* endoscopic resection

Adverse events and complications, including bleeding, perforation, aspiration pneumonitis, precordial skin burn, and procedure-related mortality, were assessed. Bleeding was defined as follows: (1) discontinuance or postponement of ER due to severe hemorrhage, (2) alteration of the endoscopic method from ESD to EMR during endoscopic treatment because of severe active hemorrhage resulting in low visibility with unstable vital signs, (3) the occurrence of melena or hematemesis, or (4) the detection of ongoing hemorrhage or the presence of coagulated blood in the stomach with apparent bleeding spots on second-look endoscopy, which was performed routinely on the day after ESD. Perforation was confirmed by observation of mesenteric fat during ESD or by detection of free air or pneumomediastinum on X-ray films or computed tomography scans. Aspiration pneumonitis was diagnosed based on the clinical findings and X-ray films. Precordial skin burns were clinically surveyed after ER during hospitalization. Procedure-related mortality was defined as death within 30 days due to complications.

### Long-term outcomes

The enrolled patients were followed up by computed tomography scan and endoscopy every six to twelve months after ER. The long-term outcomes included local recurrence, metachronous GTCs that were discovered more than one year after the initial session of ER, the rate of post-ER surgery, the 5-year overall survival (OS) rate, the disease-specific survival (DSS) rate, and the cause of death. To assess metachronous GTCs, all patients (*n* = 22) were investigated. For other long-term outcomes, a patient who was attempted ER and whose lesion could not be successfully removed endoscopically, was excluded from the analysis. The period of survival was counted starting from the date of initial ER to the date of death or the last verified date of survival.

To determine the prognostic indicators for early stage GTC treated by ER, we evaluated the clinical characteristics of the patients according to age, body mass index (BMI), prognostic nutritional index (PNI), Charlson comorbidity index (CCI), and Glasgow prognostic score (GPS).

### Statistical analysis

To estimate the factors affecting the overlooking of lesions, the relative risks were calculated. Fisher’s exact test was used to statistically analyze risk factors. The reconstruction route of gastric tube, location of GTC lesions, and tumor size were considered as possible risk factors.

Survival rates at each time point were based on Kaplan–Meier estimation. To estimate the factors affecting prognosis, hazard ratios (HRs) were calculated using the Cox proportional hazards model.

Quantitative data are expressed as medians [ranges (maximum-minimum)]. Categorical data are expressed as numbers (percentages). Statistical significance was set at *p* < 0.05.

All statistical analyses were conducted using EZR software, version 1.54 (Saitama Medical Center, Jichi Medical University, Saitama, Japan) [30].

## Results

### Patient characteristics and endoscopic findings

The clinical characteristics of the recruited patients and endoscopic findings of the lesions are shown in Table 2. A total of 22 consecutive patients with 43 lesions were treated in 42 ER sessions. All the patients were male and had a mean age of 72.3 years old at the date of initial ER for GTC, and the median period after esophagectomy was 8.2 years (range 0.2–21.9 years). Among the 22 patients, the initial GTC lesions were diagnosed in 18 patients during their annual endoscopy for esophageal cancer follow-up, in 2 patients during endoscopy performed as a routine periodic health examination, and in 2 patients during endoscopic investigation for abdominal symptoms that may not be related to GTC. Of the 22 patients, 9 (40.9%) had multiple lesions at the time of the initial ER session. However, 6 (54.5%) of 11 synchronous lesions were overlooked until the initial ER. Reconstruction of the gastric tube was retrosternal in 15 patients (68.2%) and post-mediastinum in 7 patients (31.8%). None of the patients had a pre-sternal reconstruction route. Nine (40.9%) of the patients had undergone perioperative chemotherapy for esophageal cancer. As for the location of GTC, the lower third was most frequent, followed by the middle third and upper third. In detail, GTC in the lower third of reconstructed gastric tube was predominant in post-mediastinum patients, whereas in the retrosternal patients, the proportion of GTC located in the lower third and middle third was the same. Of the 43 treated lesions, the median size was 12 mm (range, 3–66 mm), and the predominant macroscopic type was 0-IIc (48.8%), followed by 0-IIa (23.3%). Eleven (25.6%) patients had ulcerations. Regarding the histological type, 41 (95.3%) of them were differentiated adenocarcinoma. According to the Japanese Gastric Cancer Association Gastric Cancer Treatment Guidelines 2010 (ver. 3), 25 (58.1%) lesions were within the absolute indication for ER, 14 lesions (32.6%) met the expanded criteria, and 4 lesions (9.3%) that did not meet the indication criteria were also treated. The median period from esophagectomy to GTC detection in all 43 lesions was 9.0 years (range 0–21.8 years). One lesion was detected in endoscopic assessment which was performed after preoperative chemotherapy for esophageal cancer, and just before esophagectomy.

**Table 2:** Patient characteristics and endoscopic findings

Patients/lesions/ER sessions, No	22/43/42
Age at the date of initial ER, mean ± SD, years old	72.3 ± 8.0
Gender, No. (%)	
Male	22 (100%)
Female	0
Habit of daily drinking, No. (%)	14 (63.6%)
Smoking history, No. (%)	20 (90.9%)
Period from esophagectomy to initial ER for GTC, median, years (range)	8.2 (0.2–21.9)
Reason of performing examination which detected initial GTC, No. (%)	
Annual endoscopy for esophageal cancer follow-up	18 (81.8%)
Periodic health examination	2 (9.1%)
Investigation for abdominal symptoms	2 (9.1%)
Patients with multiple GTC lesions at the time of initial ER, No. (%)	9 (40.9%)
Synchronous lesions at the time of initial ER session, No	11
Detected at the same time with the initial lesion, No. (%)	5 (45.5%)
Discovered within one year after initial ER, No. (%)	6 (54.5%)
Reconstruction route of gastric tube, No. (%)	
Pre-sternal	0
Retrosternal	15 (68.2%)
Post-mediastinum	7 (31.8%)
Perioperative chemotherapy for esophageal cancer, No. (%)	
Preoperative	7 (31.8%)
Postoperative	2 (9.1%)
Location of GTC lesions, No. (%)	
Upper third	3 (7.0%)
Middle third	17 (39.5%)
Lower third	23 (53.5%)
Location of GTC lesions after retrosternal reconstruction, No. (%)	
Upper third	2 (6.7%)
Middle third	14 (46.7%)
Lower third	14 (46.7%)
Location of GTC lesions after post-mediastinum reconstruction, No. (%)	
Upper third	1 (7.7%)
Middle third	3 (23.1%)
Lower third	9 (69.2%)
Tumor size, median, mm (range)	12 (3–66)
Macroscopic type, No. (%)	
0-I	2 (4.7%)
0-IIa	10 (23.3%)
0-IIb	1 (2.3%)
0-IIc	21 (48.8%)
Combined	9 (20.9%)
Recurrence	0
Ulcer finding, No. (%)	
Absent	32 (74.4%)
Present	11 (25.6%)
Histological type, No. (%)	
Differentiated (tub1, tub2, pap)	41 (95.3%)
Undifferentiated (por, sig)	2 (4.7%)
Clinical indications for ESD, No. (%)	
Absolute	25 (58.1%)
Expanded	14 (32.6%)
Outside indications	4 (9.3%)
Period from esophagectomy to GTC detection, median, years (range)	9.0 (0–21.8)

*ER* endoscopic resection, *GTC* gastric tube cancer, *SD* standard deviation, *ESD* endoscopic submucosal dissection

Clinical characteristics of the six overlooked lesions and assessment of risk factors for overlooking the lesions are shown in Tables 3, 4. Five of the six overlooked lesions (83.3%) were located in the gastric tube reconstructed via the retrosternal route. These lesions tended to be small, and four (66.7%) of the six lesions were of the 0-IIc type. All were within the absolute or expanded criteria for ER. The location of the middle third was an estimated risk factor for overlooking the lesion (*p* = 0.028).

**Table 3:** Clinical characteristics of six overlooked lesions

Reconstruction route of gastric tube, No. (%)	
Pre-sternal	0
Retrosternal	5 (83.3%)
Post-mediastinum	1 (16.7%)
Location of GTC lesions, No. (%)	
Upper third	1 (16.7%)
Middle third	5 (83.3%)
Lower third	0
Tumor size, median, mm (range)	12 (5–22)
Macroscopic type, No. (%)	
0-I	0
0-IIa	1 (16.7%)
0-IIb	1 (16.7%)
0-IIc	4 (66.7%)
Combined	0
Ulcer finding, No. (%)	
Absent	6 (100%)
Present	0
Histological type, No. (%)	
Differentiated (tub1, tub2, pap)	6 (100%)
Undifferentiated (por, sig)	0
Clinical indications for ESD, No. (%)	
Absolute	5 (83.3%)
Expanded	1 (16.7%)
Outside indications	0

*GTC* gastric tube cancer, *ESD* endoscopic submucosal dissection

**Table 4:** Assessment of risk factors for overlooking

	Overlooked	Unoverlooked	RR	*p* value
Reconstruction route of gastric tube, No. (%)				
Retrosternal	5	26	2.097 (0.308–16.180)	0.652
Post-mediastinum	1	12		
Location of GTC lesions, No. (%)				
Middle third	5	12	7.647 (1.327–48.556)	0.028
Upper or Lower third	1	25		
Tumor size				
10 mm and smaller	3	11	2.455 (0.485–12.526)	0.364
Larger than 10 mm	3	27		

Including a metachronous lesion resected by surgery (*n* = 44)

*GTC* gastric tube cancer, *RR* relative risk

### Short-term outcomes

The short-term outcomes of ER are shown in Table 5. Within 42 sessions of ER, two different lesions were simultaneously and independently resected in one session, while only one lesion was resected for each treatment in 40 sessions, and the lesion was endoscopically unresectable in one session. Therefore, 42 out of 43 lesions were resected in 42 sessions of ER. Among those 43 lesions, 9 (20.9%) were treated using the EMR procedure, and 34 (79.1%) were treated using the ESD procedure. ER was unable to be performed in 1 of the 43 lesions because of intraoperative bleeding. There were 34 en bloc resections (79.1%), 30 en bloc resections with tumor-free margins (R0 resections, 69.8%), and 29 curative resections (67.4%) based on the Japanese Gastric Cancer Association criteria. Within 42 sessions of ER, adverse events included aspiration pneumonitis (*n* = 1, 2.4%), intraoperative bleeding (*n* = 3, 7.1%), and delayed bleeding (*n* = 4, 9.5%) without any emergency surgery or blood transfusion. There were no perforations, precordial skin burns, or procedure-related deaths.

**Table 5:** Short-term outcomes of ER

ER procedure, No. (%) (*n* = 43)	
ESD	34 (79.1%)
EMR	9 (20.9%)
ER quality, No. (%) (*n* = 43)	
En bloc resection	34 (79.1%)
R0 resection	30 (69.8%)
Curative resection	29 (67.4%)
Fractional resection	8 (18.6%)
Not resected endoscopically	1 (2.3%)
Adverse event, No. (%)	
ER sessions with any complication (*n* = 42)	8 (19.0%)
Bleeding	7 (16.7%)
Perforation	0
Aspiration pneumonitis	1 (2.4%)
Precordial skin burn	0
Procedure-related death	0

22 patients with 43 lesions, treated in 42 sessions of ER

*ER* endoscopic resection, *EMD* endoscopic mucosal resection, *ESD* endoscopic submucosal dissection

A comparison of EMR and ESD is shown in Table 6. In cases of ESD, the en bloc dissection rate was 97.1%. Regarding complications, the rates of bleeding, perforation, and aspiration pneumonitis in ESD cases were 17.6%, 0%, and 2.9%, respectively. Both the rate of en bloc resection and the rate of complications tended to be higher in ESD cases than in EMR cases.

**Table 6:** Comparison of endoscopic mucosal resection (EMR) and endoscopic submucosal dissection (ESD)

	EMR (*n* = 9)	ESD (*n* = 34)
ER quality, No. (%) (*n* = 43)		
En bloc resection	1 (11.1%)	33 (97.1%)
R0 resection	1 (11.1%)	29 (85.3%)
Fractional resection	7 (77.8%)	1 (2.9%)
Not resected endoscopically	1 (11.1%)	0
Adverse event, No. (%)		
ER sessions with any complication (*n* = 42)	1 (11.1%)	7 (20.6%)
Bleeding	1 (11.1%)	6 (17.6%)
Intraoperative/postoperative	1/0	2/4
Perforation	0	0
Aspiration pneumonitis	0	1 (2.9%)
Precordial skin burn	0	0
Procedure-related death	0	0

22 patients with 43 lesions, treated in 42 sessions of endoscopic resection (ER)

The histopathological results of the 42 lesions endoscopically resected are presented in Table 7. The median tumor size was 12 mm in the major axis, and 40 lesions (95.2%) were differentiated. Final pathology revealed 3 tumors with submucosal invasion (7.2%), 11 with ulceration (26.2%), 0 with lymphatic infiltration, and 1 lesion with vascular infiltration (2.4%), respectively. The horizontal and vertical margins were inconclusive in three (7.1%) cases each. Of the 42 endoscopically resected lesions, 29 lesions (69.0%) were judged to be curatively removed.

**Table 7:** Histopathological results (*n* = 42)

Tumor size, median, mm (range)	12 (3–90)
Histological type, No. (%)	
Differentiated (tub1, tub2)	40 (95.2%)
Undifferentiated (por, sig)	2 (4.8%)
Mixed	0
Tumor depth, No. (%)	
M	39 (92.9%)
SM1	1 (2.4%)
SM2	2 (4.8%)
MP	0
Ulcer finding, No. (%)	
Absent	31 (73.8%)
Present	11 (26.2%)
Lymphatic infiltration, No. (%)	
Negative	42 (100%)
Positive	0
Vascular infiltration, No. (%)	
Negative	41 (97.6%)
Positive	1 (2.4%)
Horizontal margin, No. (%)	
Negative	39 (92.9%)
Positive	0
Inconclusive	3 (7.1%)
Vertical margin, No. (%)	
Negative	39 (92.9%)
Positive	0
Inconclusive	3 (7.1%)
Curability of ER, No. (%)	
Curative	29 (69.0%)
Non-curative with a possible risk of LNM	6 (14.3%)
Non-curative with only HM1/HMX or fractional resection	7 (16.7%)

42 endoscopically resected specimens

*ER* endoscopic resection, *M* intramucosal, *SM1* submucosal superficial invasion, *SM2* submucosal deep invasion, *MP* muscularis propria, *LNM* lymph node metastasis, *HM1* horizontal margin positive, *HMX* horizontal margin inconclusive

### Long-term outcomes

**Fig. 1:**
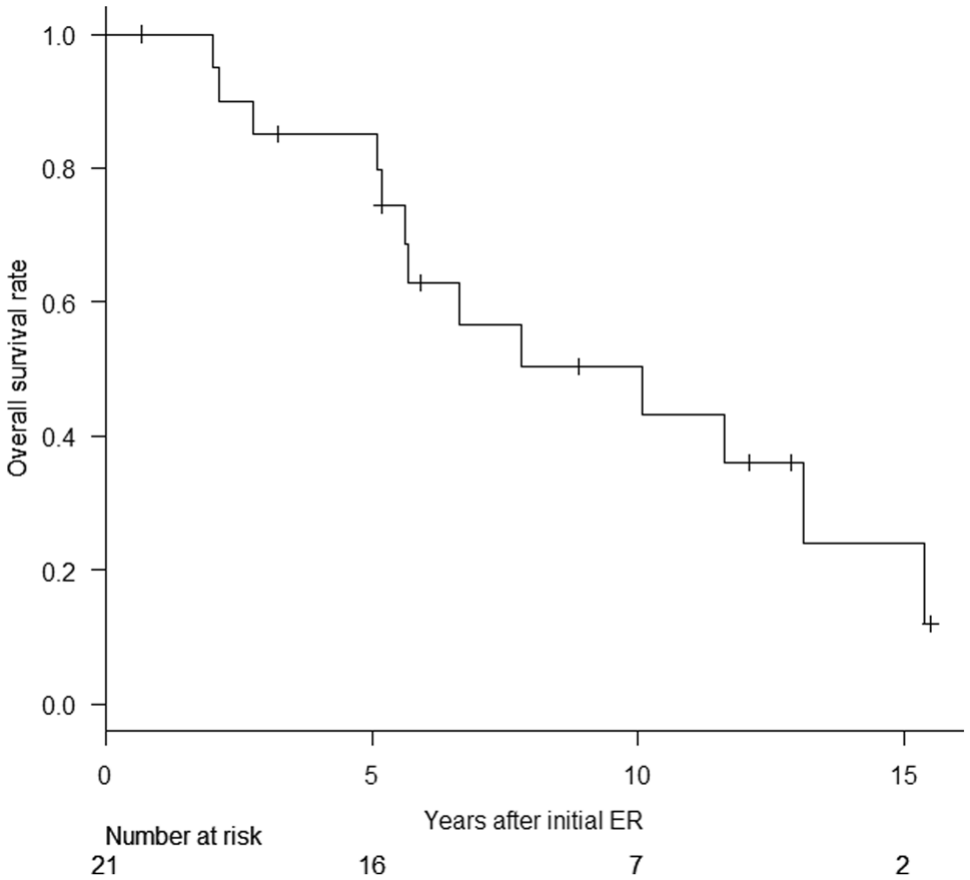
Overall survival of endoscopically resected patients (*n* = 21). Of the 21 endoscopically resected patients, 13 (61.9%) died during a median follow-up period of 5.9 (0.7–15.5) years after initial ER. The 5-year overall survival rate was 85.0%. *ER* endoscopic resection

The long-term outcomes after the initial ER are shown in Table 8. The survival curves are shown in Fig. 1.

**Table 8:** Long-term outcomes after initial endoscopic resection (ER)

Follow-up period, median, years (range)*	5.9 (0.7–15.5)
Local recurrence, No. (%)*	1 (4.8%)
Patients with multiple GTC, No. (%)#	12 (54.5%)
2 GTCs during life span	7 (31.8%)
3 GTCs during life span	2 (9.1%)
4 GTCs during life span	2 (9.1%)
5 GTCs during life span	1 (4.5%)
Patients with metachronous GTC, No. (%)#	6 (27.3%)
1 metachronous GTC	3 (13.6%)
2 metachronous GTCs	1 (4.5%)
3 metachronous GTCs	2 (9.1%)
Surgery for GTC after ER, No. (%)*	
Preventive additional surgery after non-curative ER	0
Surgery for local recurrence	1 (4.8%)
Surgery for metachronous GTC	1 (4.8%)
Death during follow-up period, No. (%)*	13 (61.9%)
5-year survival rate, %*	
Overall survival rate	85.0%
Disease-specific survival rate	100%
Causes of death, No. (%)*	
Esophageal cancer recurrence	3 (23.1%)
Primary GTC or recurrence	1 (7.7%)
Metachronous GTC	0
Other organ malignancy	3 (23.1%)
Benign disease	2 (15.4%)
Unknown	4 (30.8%)

*Except one unresected case (*n* = 21)

^#^Of all recruited patients (*n* = 22)

*ER* endoscopic resection, *GTC* gastric tube cancer

Local recurrence was detected in 1 (4.8%) of the 21 endoscopically resected patients, and metachronous GTCs were identified in 8 (36.4%) of the 22 recruited patients. Among the 21 endoscopically resected patients, 13 (61.9%) died during a median follow-up period of 5.9 years (0.7–15.5 years) after initial ER. One patient (7.7%) died of GTC recurrence, 15.4 years after the initial non-curative ER date, which was much lower than the three patients (23.1%) who died of esophageal cancer recurrence and the three patients (23.1%) who died of other organ malignancies. The 5-year OS rate was 85.0%, and the 5-year DSS rate was 100%. Of the 22 recruited patients, 12 (54.5%) experienced synchronous and/or metachronous multiple GTCs during their life span. As for malignancies other than esophageal cancer and GTC discovered during their lifetime, pharyngeal, lung, colorectal, and prostate cancers were discovered in two (9.1%), three (13.6%), one (4.5%), and two (9.1%) patients, respectively.

**Fig. 2:**
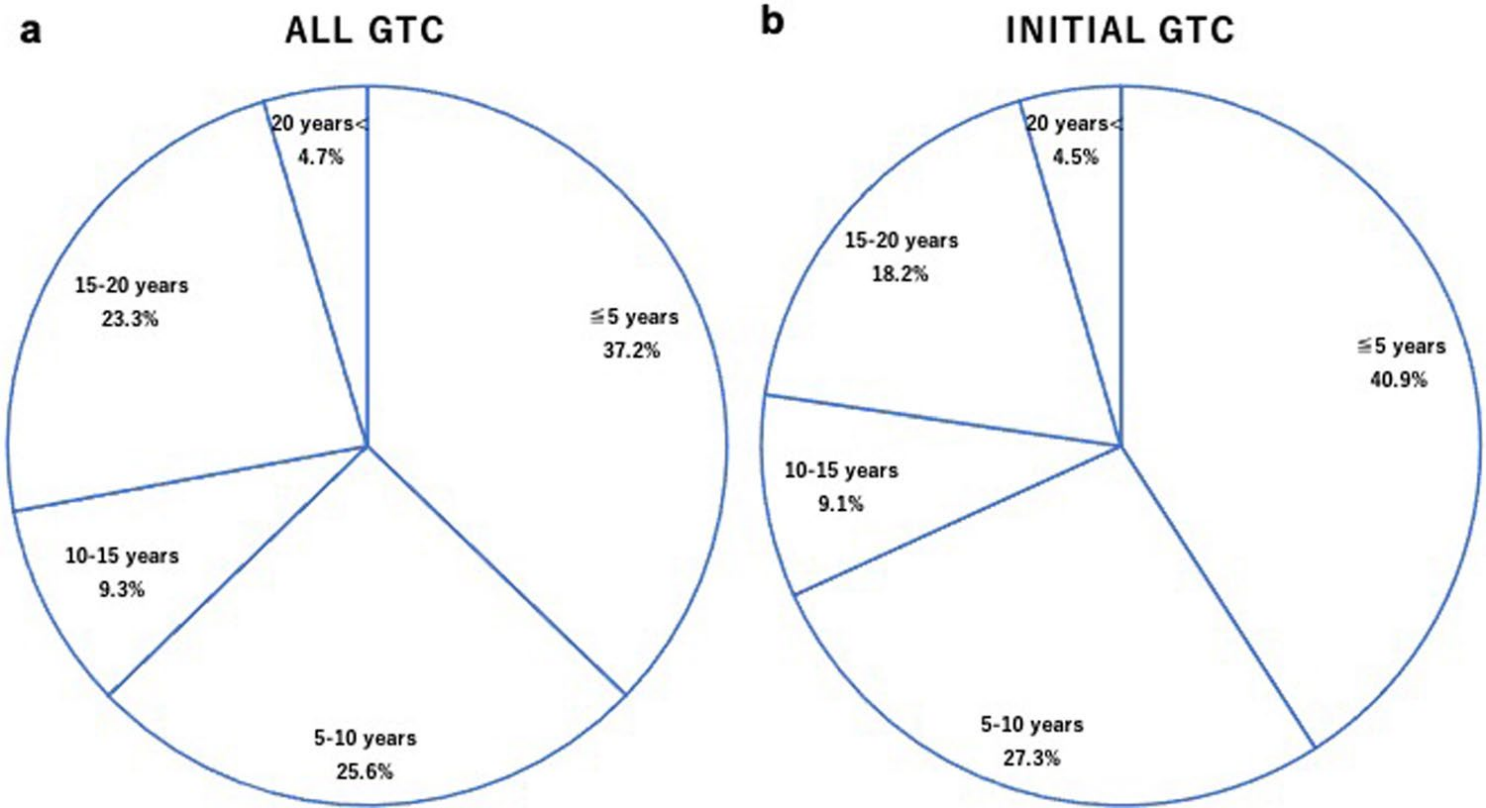
**a** The time points of GTC detection after esophagectomy (*n* = 43). ≤ 5 years: detected within 5 years, 5–10 years: detected within 5–10 years (exclusive of 5 years), 10–15 years: detected within 10–15 years (exclusive of 10 years), 15–20 years: detected within 15–20 years (exclusive of 15 years), 20 years < : detected more than 20 years after esophagectomy. Of the 43 lesions, 16 (37.2%) were detected within 5 years. In contrast, 18 lesions (41.9%) were detected more than 10 years after esophagectomy, and 2 lesions (4.7%) were detected more than 20 years after esophagectomy. *GTC* gastric tube cancer. **b** The time points of initial GTC detection after esophagectomy (*n* = 22). ≤ 5 years: detected within 5 years, 5–10 years: detected within 5–10 years (exclusive of 5 years), 10–15 years: detected within 10–15 years (exclusive of 10 years), 15–20 years: detected within 15–20 years (exclusive of 15 years), 20 years < : detected more than 20 years after esophagectomy. The rate of initial GTC discovered within 5 years after esophagectomy was only 40.9% (9/22). *GTC* gastric tube cancer

The time points for GTC detection after esophagectomy are shown in Fig. 2. 16 of 43 lesions (37.2%) were found in 9 of the 22 patients (40.9%) enrolled within five years after esophagectomy. In contrast, 18 (41.9%) lesions were detected more than 10 years after esophagectomy, and 2 (4.7%) lesions were detected more than 20 years after esophagectomy.

### Prognostic indicators

The assessment of prognostic indicators is presented in Table 9. The curability of ER, age, BMI, PNI, CCI, and GPS were not significantly related to prognosis by multivariate analysis.

**Table 9:** Assessment of prognostic indicators (*n* = 21)

	Median	Range	Multivariate analysis		
			HR	95% CI	*[* value
Curability					
Non-curative with possible LN metastasis			0.546	0.073–4.088	0.556
Clinical characteristics of the patients*					
Age, years old	71.6	56.8–85	1.005	0.998–1.012	0.148
BMI	18.7	15.4–23.1	0.836	0.659–1.06	0.140
PNI	48	43–60.8	0.916	0.787–1.066	0.257
CCI	1	0–3	1.973	0.910–4.277	0.085
GPS	0	0–1	2.328	0.553–9.808	0.249

*At the date of initial ER

*HR* hazards ratio, *CI* confidence interval, *LN* lymph node, *Alb* albumin, *BMI* body mass index, *PNI* prognostic nutritional index, *CCI* Charlson comorbidity index, *GPS* Glasgow prognostic score

## Discussion

GTCs have been discovered in 0.5–6.3% of patients after surgical esophagectomy [18, 22, 31]. Surgical resection has been considered the standard treatment for GTC, but its high mortality rate of 23.8–30% is a severe problem to be solved [18, 32, 33]. The indications for ER for EGC are described in the Japanese Gastric Cancer Association Gastric Cancer Treatment Guidelines 2010 (ver. 3) [29]. The indications were recently expanded to cover lesions with a negligible risk of lymph node metastasis [34–36] and also adapted to GTC [19–22].

To date, there are only three reports of ER for GTC with more than 30 lesions, the follow-up period was very short (1.8–3.8 years), and there was no comparison between EMR and ESD [18, 26, 27]. This is the first report of ER for GTCs comprising a large number of lesions (more than 30) to assess the long-term natural course after ER, with a median follow-up period of 5.9 years and to compare EMR and ESD for GTC from the perspective of complications and successful resection.

In our study, we did not experience any severe complications of procedure-related deaths, emergency operations, or blood transfusions. The rates of bleeding and aspiration pneumonitis showed that ESD tended to have a higher risk of complications than did EMR. However, we preferentially selected ESD over EMR because ESD has the potential to achieve a high rate of en bloc resection. Indeed, the rate of en bloc resection in our study was 97.1%, which was as high as the rate of 98% in regular ESD for EGC reported by Oda et al. [37]. On the other hand, the rate of successful en bloc resection in EMR cases was only 11.1%. However, only one patient had local recurrence in our study. Although ESD is an attractive procedure that achieves better short-term outcomes than EMR, we should emphasize that ESD for GTC is a very difficult procedure.

Among the three previous reports containing more than 30 GTCs resected by ESD, the rates of intra- or postoperative bleeding and intraoperative or delayed perforation ranged from 0% to 6.3% and 2.1% to 6.3%, respectively [18, 26, 27]. Compared to these reports, the rate of bleeding in the present study was as high as 17.6%. Conversely, there were no perforations in our study. The reason for the discrepancy between other studies and our study is unclear, but one possibility for the high bleeding rate is that as we performed endoscopic screening examinations routinely on the next day of ER, our study might have detected asymptomatic minor hemorrhages that may have been overlooked in other studies. Regarding the incidence of complications of ESD for EGC performed on unresected stomachs, Lin et al. reported that the rates of bleeding and perforation were 2.9% and 1.1%, respectively, in their meta-analysis of nine previous studies of gastric ESD [38]. Considering these previous reports and the present study, we must recognize that ESD for GTC may have a higher risk of intraoperative bleeding than regular ESD for EGC. Therefore, we strongly recommend that the procedure should be performed by highly experienced endoscopists during the dissection of GTC. Additionally, we recommend performing endoscopy routinely in all patients on the day after ESD for GTC to detect minor postoperative bleeding or exposed vessels on the ulcer bed without any symptoms and prevent severe delayed hemorrhage.

It is well known that multiple primary cancers in other organs frequently occur in esophageal cancer patients. The incidence rate of metachronous malignancies in other organs, especially squamous cell carcinoma, has been reported to range from 11.3% to 12.0% [39, 40]. Gastric cancer, including GTC, and head and neck cancer are commonly identified [39–44]. The incidence of GTC after esophagectomy is reported to be 1.3–6.3% [9, 22–24, 32]. However, there are few reports of a large number of patients with metachronous GTCs after ER of initial GTC. Nonaka et al. reported that metachronous GTCs developed in 18 (35.3%) of 51 patients who had undergone ESD for GTC during a median follow-up period of 3.8 years [18]. In the present study, we followed the patients as long as 5.9 years after the initial ER, and surprisingly, demonstrated that metachronous GTCs were discovered in 54.5% of the patients. Our results and those of the previous report show that the incidence of metachronous GTCs was remarkably higher than that of metachronous EGCs occurring in the unresected stomach, with a 3-year cumulative incidence rate of 5.9% reported by Nakajima et al. [45]. We must realize that once GTC occurs, the patients have a considerable risk of metachronous GTCs. In our study, 27 (62.8%) of 43 lesions were discovered more than five years after esophagectomy. In addition, 18 (41.9%) and 2 (4.7%) lesions were detected at more than 10 and 20 years after esophagectomy, respectively. Therefore, long-term follow-up, ideally annual endoscopic examination for an extended duration, is required after esophagectomy, especially in patients who have developed GTC at least once.

However, detecting cancer in reconstructed gastric tubes is difficult, as food often remains in the gastric tube, and gastric tubes are slim and moving all the time under the influence of the heartbeat. In fact, although there were supposed to be 11 synchronous lesions at the time of the initial ER session in our study, only 5 (45.5%) were detected at the same time as the initial lesion, indicating that 6 (54.5%) lesions were overlooked. Four of them were 0-IIc type, one was 0-IIa type, and one was 0-IIb type. In the present study, location in the middle third of the gastric tube, which is continuously affected by heartbeat, was revealed to be a risk factor for overlooking the lesion. Moreover, five of the six overlooked lesions (83.3%) were located in the gastric tube reconstructed by the retrosternal route, in which the gastric tube came close to the heart. These results indicate that instability due to adjacent heartbeats is a major factor for overlooking lesions. To prevent overlooking of GTC, attention must be paid to the small depression in the middle third of the gastric tube. Furthermore, efforts should be made to reduce food residue, using an antispasmodic agent if possible, and the examination should be performed by an expert endoscopists. The use of indigo carmine dye also seems useful for diagnosing early stage GTC, as reported previously [22].

Regarding long-term outcomes of ER for EGC, local recurrence was observed in one lesion that was intended to resect by ESD. This lesion was judged as an outside indication for ER according to the Japanese Gastric Cancer Association Gastric Cancer Treatment Guidelines 2010 (ver. 3), and resected endoscopically after explanation to the patient. During the endoscopic treatment, severe intraoperative bleeding occurred, and the procedure was converted from ESD to fractional EMR. Histological examination of the resected specimen revealed ulcerative findings and invasion of tumor cells into the submucosal layer at a depth of more than 500 μm. The patient was followed carefully, and local recurrence was detected seven months after treatment. The patient underwent surgical resection of the reconstructed gastric tube one month after the diagnosis of recurrence. However, he finally died of GTC recurrence 9.5 years after surgery. Except for this patient, no patient died of GTC in our study. We believe that our attentive follow-up of the patients for a long period after initial ER contributed to the early detection of metachronous EGCs, resulting in a good prognosis.

Of the 21 successfully resected patients, 13 (61.9%) died during the median follow-up period of 5.9 years. The most common cause of death was malignancy in other organs, including esophageal cancer recurrence. It is well known that gastric cancer, including GTC, and head and neck cancer are commonly identified in esophageal cancer patients as a multiple cancer [39]. However, we demonstrated that lung cancer (13.6%) was the most commonly identified cancer in recruited patients, followed by pharyngeal cancer (9.1%), prostate cancer (9.1%), and colorectal cancer (4.5%). On the other hand, all patients were male in this study, which is consistent with the fact that esophageal cancer is predominant in males, with a male–female ratio of approximately 6:1. Risk factors for esophageal cancer in Japan are habits of alcohol intake and smoking, and both of these habits are frequently observed more in the male population [46]. We speculate that the development of lung cancer, pharyngeal cancer, and colorectal cancer in our patients is strongly influenced by their habit of smoking and drinking, similar to esophageal cancer. Therefore, it is very important to educate patients not to smoke or drink, in addition to detecting malignancies in other organs as early as possible, to improve prognosis after ER for GTC.

The strength of this study lies in its long-lasting follow-up of patients, with an acceptable number of cases compared to similar studies. Our study sheds light on the surveillance after ER. A limitation of our study is that it was a retrospective study. Moreover, we have only a limited number of cases, although relatively large numbers compared to previous reports, as this is a single-center study, and ER for GTCs is rare; our results may have been influenced by selection bias. Therefore, a multicenter prospective trial is needed to obtain further knowledge.

In conclusion, ER for GTC is feasible without severe adverse events. In particular, ESD is permissible in terms of R0 resection for treating early-stage GTC. However, bleeding occurred more frequently than in regular ESD for EGC. Therefore, meticulous preventive endoscopic hemostasis is recommended. We also recommend routine endoscopy on the day after ESD for early detection of minor bleeding or exposed vessels on the ulcer bed without any symptoms of the patient. To improve prognosis, early detection of metachronous GTC and other organ malignancies is crucial. Lifelong follow-up considering multiple cancers of other organs and metachronous GTCs is essential. To prevent overlooking GTC lesions, attentive endoscopic observation of the middle third of the gastric tube is required.
